# NRF1 coordinates mitochondrial adaptations to dampen intracellular ROS and inflammatory responses during ischemia reperfusion

**DOI:** 10.1038/s41420-025-02461-5

**Published:** 2025-05-15

**Authors:** Jiakun Li, Jiawei Yan, Guowei Tu, Wenjiao Jiang, Yue Qiu, Ying Su, Changhong Miao, Zhe Luo, Tiffany Horng

**Affiliations:** 1 Department of Anesthesiology, Zhongshan Hospital, Fudan University, Shanghai, China; 2 Shanghai Institute of Infectious Disease and Biosecurity, Fudan University, Shanghai, China; 3 School of Life Sciences and Technology, ShanghaiTech University, Shanghai, China; 4 Cardiac Intensive Care Center, Zhongshan Hospital, Fudan University, Shanghai, China

**Keywords:** Cytokines, Toll-like receptors

## Abstract

Ischemia reperfusion injury (IRI) is commonly seen in surgical procedures involving cardiopulmonary bypass and post-shock reperfusion. Sudden restoration of blood flow after a period of ischemia triggers a rapid accumulation of reactive oxygen species (ROS) and oxidative stress that promote pathological injury. Macrophage-derived inflammatory responses are also thought to contribute to such injury, but how ROS influences tissue macrophages and their elaboration of inflammatory cytokines in IRI remains poorly understood. In this study, we showed that macrophages mobilize mitochondrial adaptations during reoxygenation, including mitochondrial fission and ubiquitin proteasome system (UPS) flux. Furthermore, the transcription factor Nuclear Factor Erythroid 2 Like 1 (NRF1) is rapidly induced during reoxygenation in response to rising levels of ROS. Induction of NRF1 upregulates ubiquitin proteasome system (UPS) and mitophagy pathways to mediate mitochondrial fusion/fission dynamics and dampen ROS production, allowing for alleviation of oxidative stress and the inflammatory response. Conversely, the absence of myeloid NRF1 leads to increased ROS, driving enhanced inflammation and kidney injury in a mouse model of IRI. We thus identify macrophage NRF1 as a master regulator of mitochondrial homeostasis, antioxidant defense, and inflammatory responses in IRI.

## Introduction

Ischemia reperfusion injury (IRI) occurs when blood flow is suddenly restored after a period of ischemia, commonly seen in surgical procedures involving cardiopulmonary bypass and post-shock reperfusion [1]. Such injury is associated with metabolic dysfunction and structural damage to the tissue, and has a complex etiology. Increasing evidence indicates that excessive production of reactive oxygen species (ROS) and oxidative stress are the main pathological processes driving tissue reperfusion injury [2]. Oxidative stress disrupts cellular redox balance, and triggers mitochondrial dysfunction, which further amplifies ROS production. For example, recent studies indicate that the TCA metabolite succinate accumulates during ischemia, and upon reperfusion, drives ROS production through reverse electron transport to induce cellular and tissue damage [3].

Macrophages, as innate immune cells, initiate the inflammatory response during tissue IRI. Early in the injury response, macrophages are activated to release pro-inflammatory cytokines that recruit and activate cells of the adaptive immune system, thereby orchestrating the development and progression of the inflammatory responses in IRI tissues [4]. In particular, interleukin-6 (IL6) secreted by such activated macrophages not only contributes to tissue injury but also serves as an important biomarker of early inflammation and of tissue injury [5, 6]. Of note, existing research has mainly focused on the effects of IRI on tissue damage, and there remains a dearth of studies on the impact of IRI on macrophages. Understanding the mechanisms by which IRI affects macrophages and identifying protective factors of this process could have significant therapeutic and preventative implications [7].

Mitochondria play a prominent role during oxidative stress. In addition to being a major source of ROS, oxidatively damaged, dysfunctional mitochondria are also linked to the exacerbation of inflammatory responses, especially in macrophages. It is therefore not surprising that adaptive mechanisms centered on mitochondria are induced during oxidative stress. In particular, disturbances in mitochondrial function like increased mitochondrial ROS or loss of mitochondrial membrane potential can trigger mitophagy. At dysfunctional mitochondria, ubiquitination of mitochondrial proteins is initiated by the PINK-PARKIN pathway to engage mitophagy receptors like FUNDC1 and BNIP3L, leading to the clearance of damaged mitochondria by mitophagy [8–11]. Interestingly, recent studies have also indicated a link between such ubiquitination and proteasomal degradation. PARKIN-mediated ubiquitination of mitofusins, key regulators of mitochondrial fusion, targets them for degradation by the proteasome, which apparently is a prerequisite for mitophagy [12, 13].

NRF1 is a member of the nuclear factor erythroid 2-like (NFE2L) transcription factor family. This family of transcription factors, which also includes NRF2 and NRF3, regulate the expression of genes containing antioxidant response elements (AREs) in their promoter regions [14, 15]. Subsequent to its role in regulating antioxidant defense, NRF1 was found to be a master regulator of proteasome subunit gene expression [16]. In brown fat, NRF1 is induced by cold adaptation to increase proteasome activity, thus promoting mitochondrial metabolism and heat production [17]. NRF1 is synthesized in a latent form that is tethered to the cytosolic face of the ER membrane, and requires proteolytic processing to be released from the ER. mTORC1 signaling has been reported to be an upstream regulator of NRF1, triggering its release from the ER prior to nuclear entry and induction of proteasome subunit genes [18].

In this study, we identify the transcription factor NRF1 (encoded by NFE2L1), as an inducible component of the macrophage response to oxidative stress during IRI. Induction of NRF1 promotes mitochondrial adaptations to curb inflammatory responses, and is thus a key protective mechanism during IRI.

## Results

### A mitochondrial fission-ROS axis influences macrophage inflammatory cytokine production during OGD/R

**Fig. 1:**
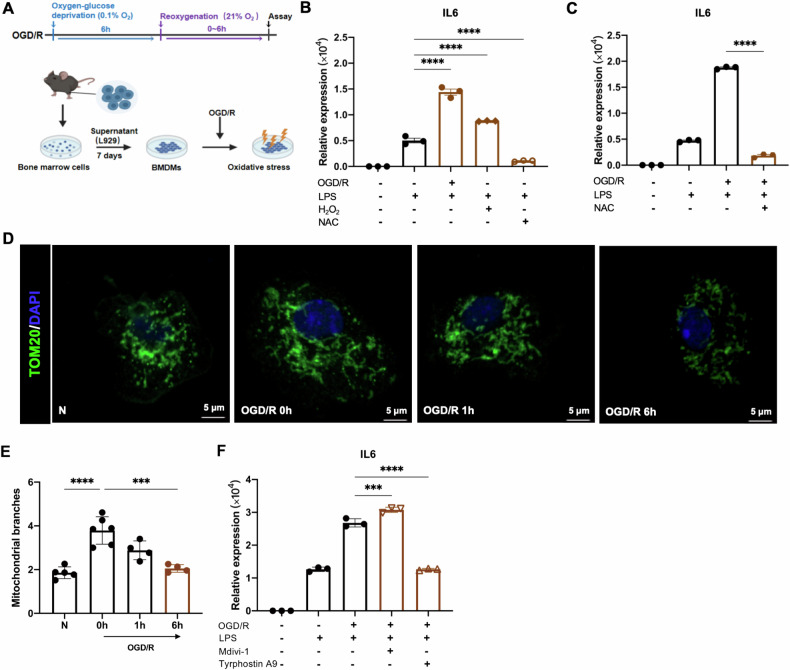
A mitochondrial fission-ROS axis regulates inflammatory gene induction during OGD/R. **A** In vitro model of oxygen-glucose deprivation and reoxygenation in BMDMs. **B**, **C** BMDMs were treated with OGD/R, LPS (10 ng/mL), H_2_O_2_ (150 μM) and NAC (10 μM) as indicated, followed by analysis of Il6 gene expression by qRT-PCR. **D**, **E** TOM20 staining of BMDMs subject to OGD followed by different durations of reoxygenation was visualized by immunofluorescent confocal microscopy. Scale bar = 5 μm. Representative image in (**D**); analysis and quantitation by Image J in (**E**). **F** BMDMs were treated with OGD/R, LPS (10 ng/mL), Mdivi-1 (30 μM), and Tyrophostin A9 (10 μM) as indicated, followed by analysis of Il6 gene expression by qRT-PCR. Statistical test by one-way ANOVA and 2-tailed samples t-test (*n* = 3–6 and representative of 3–6 independent experiments, n.s. no significance, *P < 0.05, **P < 0.01, ***P < 0.001, ****P < 0.0001).

Ischemia-reperfusion injury (IRI) is a common tissue injury in clinical practice. In vitro models simulating IRI are relatively mature, primarily involving hydrogen peroxide treatment or glucose-oxygen deprivation and reoxygenation models [19]. We utilized the OGD/R model to simulate effects of IRI on macrophages (Fig. 1A). We found that although OGD/R and hydrogen peroxide (H_2_O_2_) treatment did not directly induce the expression of the macrophage inflammatory marker IL-6 (commonly used clinically as an early inflammation indicator) (Supplementary Fig. 1), OGD/R synergized with the microbial product lipopolysaccharide (LPS) to enhance the induction of *Il6* transcript in bone marrow derived macrophages (BMDMs) (Fig. 1B). ROS has been shown to promote macrophage inflammatory responses [20], and consistently, H_2_O_2_ treatment synergized with LPS stimulation to induce Il6 transcript, while the ROS scavenger N-Acetylcysteine (NAC) diminished LPS-inducible Il6 (Fig. 1B). Therefore, and given the prominent production of ROS during IRI, we hypothesized that the ability of OGD/R to synergize with LPS stimulation (Fig. 1B) was mediated by its induction of ROS. In support of this idea, NAC treatment attenuated the ability of OGD/R to synergize with LPS stimulation to induce Il6 (Fig. 1C).

We next asked about the source of the ROS that synergized with LPS stimulation during OGD/R. Mitochondria are an important source of ROS, especially during IRI. As a starting point to examine the mitochondria of macrophages subject to OGD/R, we analyzed mitochondrial morphology by staining with the mitochondrial marker TOM20. We found that during OGD/R, the mitochondrial network underwent dynamic changes. Initially, oxygen and glucose deprivation forced the mitochondria to shift to a hyperfused phenotype, as indicated by increased mitochondrial branch number, while reoxygenation elicited a gradual, time-dependent reduction of mitochondrial branch number, indicating a return towards more fizzed mitochondria (Fig. 1D, E). The initial hyperfusion is consistent with previous studies indicating that mitochondrial fusion is an adaptive response elicited during nutrient deprivation to maximize ATP production, while mitochondrial fission is known to lower mitochondrial membrane potential to limit ROS production [21–24]. Therefore, the dynamic shift of the mitochondria from fusion to fission (Fig. 1D, E) may be a mechanism to limit oxidative stress during OGD/R. Indeed, we found that treatment with Mdivi-1, which promotes mitochondrial fusion, modestly but significantly enhanced Il6 induction during OGD/R, while treatment with Tyrphostin A9, which promotes mitochondrial fission, robustly reduced such induction (Fig. 1F). Together, these findings indicate that mitochondrial fission is dynamically regulated during reoxygenation, and contributes to a dampening of ROS and inflammatory responses.

### Induction of macrophage NRF1 during OGD/R is mediated by the mTORC1 signaling pathway

**Fig. 2:**
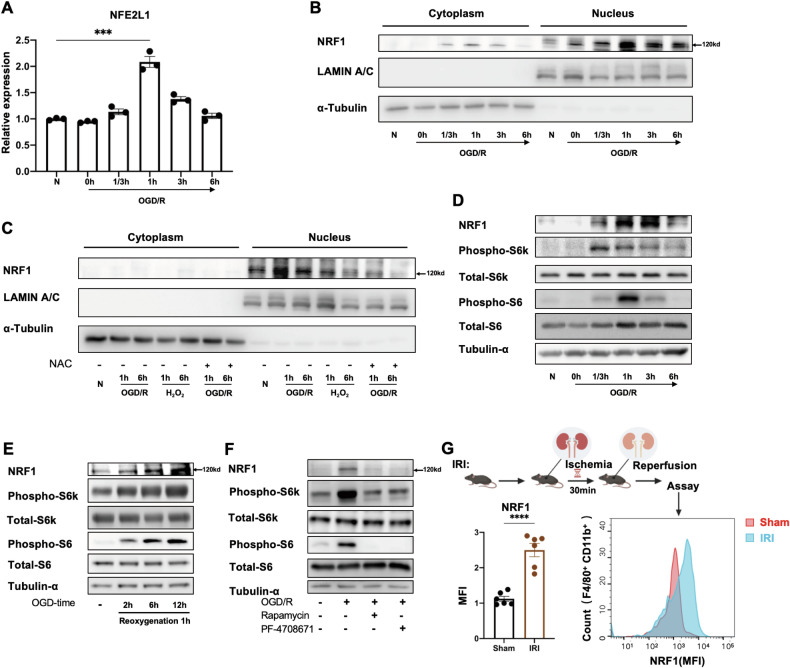
mTORC1 signaling induces NRF1 during OGD/R. **A**, **B** BMDMs were treated with OGD followed by different durations of reoxygenation. Nfe2l1 transcript was analyzed by qRT-PCR (**A**) and NRF1 proteins levels in cytosolic and nuclear fractions was analyzed by WB (**B**). **C** BMDMs were treated with OGD followed by different durations of reoxygenation +/− H_2_O_2_ treatment during reoxygenation. NRF1 proteins levels in cytosolic and nuclear fractions was analyzed by WB. **D** BMDMs were treated with OGD followed by different durations of reoxygenation. NRF1 protein levels and mTORC1 signaling were assessed by WB. **E** BMDMs were subject to different durations of OGD followed by 1 h reoxygenation. NRF1 levels and mTORC1 signaling were assessed by WB. **F** BMDMs were subject to OGD/R +/− mTOR inhibitor (rapamycin, 400 nM) or S6K inhibitor (PF-4708671, 10 μM) during reoxygenation as indicated. NRF1 levels and mTORC1 signaling were assessed by WB. **G** *Top*, mouse model of ischemia and reperfusion injury induced acute kidney injury (IRI-AKI). *Bottom*, NRF1 MFI in F4/80^+^ CD11b^+^ cells of kidneys subject to IRI-AKI or sham; quantitation and representative histogram are shown. Statistical test by one-way ANOVA and 2-tailed samples t-test (*n* = 3–6 and representative of 3–6 independent experiments, n.s. no significance, *P < 0.05, **P < 0.01, ***P < 0.001, ****P < 0.0001).

Next, we turned our attention to the nuclear factor erythroid 2-like (NFE2L) family of transcription factors. We found that the transcript levels of Nfe2l2 (NRF2) and Nfe2l3 (NRF3) were not induced during OGD/R (Supplementary Fig. 2A), while that of NFE2L1 (NRF1) increased during early reoxygenation, reaching a peak at 1 h (Fig. 2A). NRF1 is normally expressed in an ER-anchored form, and upon proteolytic processing, translocates to the nucleus where it is active as a transcription factor to upregulate expression of various genes including its own gene. Isolating nuclear and cytosolic fractions from BMDMs subject to OGD/R, we found that NRF1 protein levels, especially those in the nucleus, increased during reoxygenation. The increase was visible at 20 m, peaked at 1 h, and gradually decreased after 6 h (Fig. 2B, Supplementary Fig. 2B).

We next considered how NRF1 could be activated during OGD/R. We initially hypothesized that ROS could play a role, because of the increases in ROS during OGD/R. Indeed, although H_2_O_2_ treatment alone seemed to have minimal effects on NRF1 induction, NAC treatment during OGD/R abrogated NRF1 induction (Fig. 2C, Supplementary Fig. 2C). Therefore, ROS production during OGD/R was indispensable for NRF1 induction.

mTORC1 is a major metabolic hub that links nutrient availability to the induction of anabolic metabolism [25–27]. In addition, mTORC1 and its upstream regulator AKT are regulated by intracellular levels of ROS, suggesting that mTORC1 may play a role in maintaining oxidative balance within cells [28]. mTORC1 activity has also been reported to promote NRF1 induction, leading to NRF1-mediated upregulation of proteasome subunit genes [29]. Investigating the potential existence of a mTORC1-NRF1-proteasome axis in macrophages during OGD/R, we examined the activation of the mTORC1 signaling pathway using phosphorylation of S6K and S6, downstream targets of mTORC1, as readouts. We found that mTORC1 signaling was rapidly induced during OGD/R, peaking within an hour (Fig. 2D, Supplementary Fig. 2D). These findings are consistent with previous reports of ROS-dependent upregulation of mTORC1 activity, and with the kinetics of NRF1 induction that we observed (Fig. 2A–E). We next subjected BMDMs to different durations of oxygen-glucose deprivation (OGD) (2 h, 6 h, and 12 h) followed by reoxygenation to induce oxidative stress. Interestingly, we found that the extent of mTORC1 and NRF1 activation increased commensurate with the duration of oxygen and glucose deprivation (Fig. 2E, Supplementary Fig. 2). Finally, we used Rapamycin and PF-4708671, specific inhibitors of mTORC1 and S6K respectively, to determine a role for mTORC1 in NRF1 induction during OGD/R. We found that these inhibitors strongly reduced NRF1 induction, concomitant with inhibition of mTORC1 signaling (Fig. 2F, Supplementary Fig. 2F).

To determine NRF1 expression in macrophages during ischemia-reperfusion injury, we established a C57BL/6 J mouse model of acute kidney injury induced by ischemia and reperfusion injury (Fig. 2G). Compared to counterparts in the sham operation group, NRF1 expression was significantly upregulated in CD11b^+^F4/80^+^ kidney macrophages in the IRI group (Fig. 2G).

Thus, macrophage NRF1 is induced by mTORC1 signaling and perhaps also by oxidative stress in OGD/R. Furthermore, the degree of activation of the mTORC1-NRF1 axis seems to be commensurate with the degree of oxidative stress.

### UPS induction during OGD/R may preserve mitochondrial functionality

**Fig. 3:**
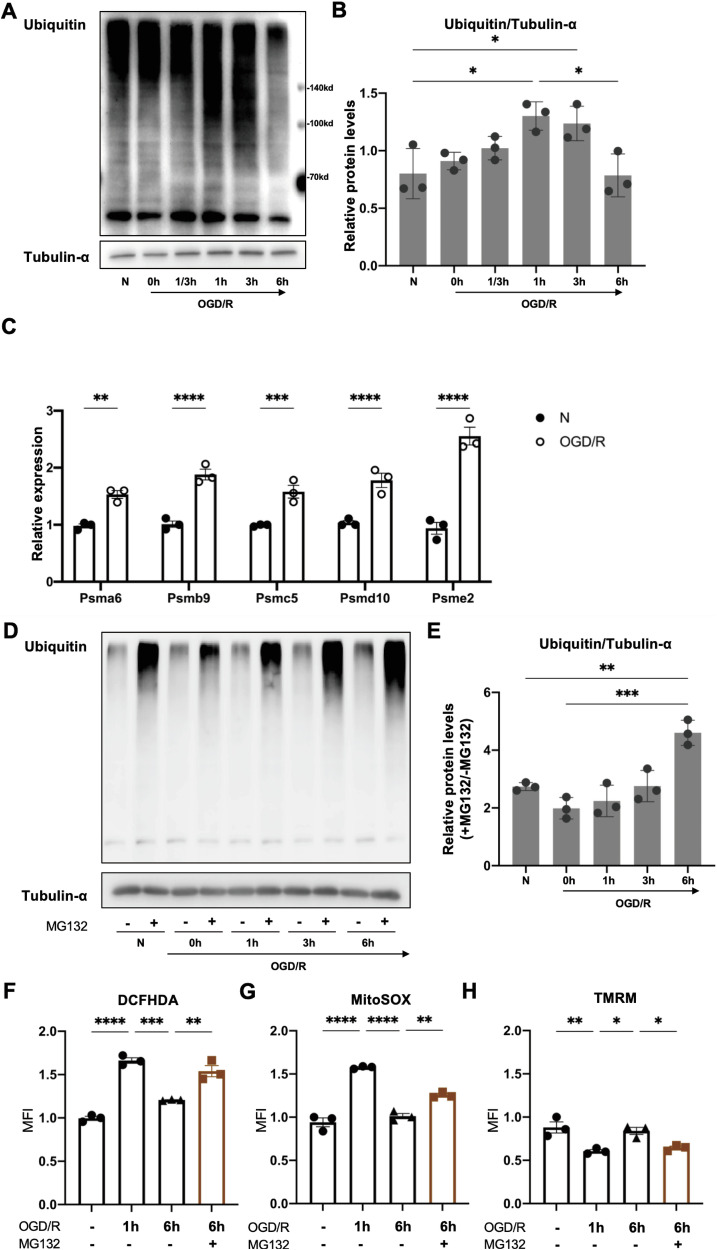
UPS induction during OGD/R preserves mitochondrial functionality. **A**, **B** BMDMs were subject to different durations of OGD/R followed by analysis of poly-ubiquitinated proteins by WB(A). Ubiquitin protein levels were normalized to α-Tubulin (**B**). **C** BMDMs subject to OGD/R were analyzed for the expression of proteasome subunit genes by qRT-PCR. D-E. BMDMs subject to different durations of OGD/R were treated with MG132 (5 nM) in the last 1 h before harvest, followed by analysis of poly-ubiquitinated proteins by WB (**D**). Data are presented as fold induction of band intensity in the +MG132 condition versus the -MG132 condition for the corresponding OGD/R stimulation time point (**E**). **F**–**H** BMDMs subject to different durations of OGD/R were treated with MG132 during reoxygenation. DCFHA, MitoSox and TMRM staining was assessed by flow cytometry and MFI shown normalized to the untreated condition. Statistical test by one-way ANOVA and 2-tailed samples t-test (*n* = 3 and representative of 3 independent experiments, n.s. no significance, *P < 0.05, **P < 0.01, ***P < 0.001, ****P < 0.0001).

Oxidative stress can damage proteins, leading to protein aggregation and misfolding as well as loss of protein functionality, while clearance of damaged proteins during oxidative stress is an adaptive mechanism. NRF1 is known to be a master regulator of proteasome subunit gene expression, prompting us to examine the UPS in macrophages subject to OGD/R. First, we examined levels of poly-ubiquitinated proteins, substrates of the proteasome, during OGD/R. We found that such proteins began to accumulate 20 min after reoxygenation and peaked at around 3 h after reoxygenation (Fig. 3A, B). Next, examining expression of several proteasome subunit genes (Psma6, Psmb9, Psmc5, Psmd10 and Psme2), we found that their expression was increased during OGD/R (Fig. 3C). Such increase suggested that protein degradation in the UPS may be upregulated during OGD/R. To accurately monitor flux in the UPS, we added the proteasome inhibitor MG132 during reoxygenation, and assessed the accumulation of poly-ubiquitinated proteins by immunoblotting analysis. We found that compared to untreated BMDMs, oxygen-glucose deprivation reduced UPS flux, as indicated by diminished accumulation of poly-ubiquitinated proteins, while reoxygenation stimulated a gradual restoration of UPS flux (Fig. 3D, E). Therefore, reoxygenation is associated with a dynamic increase in proteasome gene expression and protein turnover in the UPS pathway.

A previous study showed that in BAT, mitochondrial oxidative stress induced by thermogenic activity is limited by NRF1-mediated induction of the UPS [17]. This led us to hypothesize that in macrophages subject to OGD/R, induction of the UPS may confer protection against mitochondrial oxidative stress. Indeed, we found that OGD/R was associated with a transient increase in cellular and mitochondrial ROS and drop of mitochondrial membrane potential. The changes were evident at 1 h following reoxygenation but were largely gone by 6 h (Fig. 3F–H), suggesting that during reoxygenation, adaptive mechanisms are upregulated to counter mitochondrial ROS production and preserve mitochondrial functionality. Importantly, treatment with MG132 during reoxygenation increased cellular and mitochondrial ROS levels and reduced TMRM (Fig. 3F–H). Together, these findings indicate that during reoxygenation, proteasome activity is induced and may play a role in limiting ROS production to maintain mitochondrial functionality.

### NRF1 deficiency leads to increased mitochondrial fusion that promotes ROS production and mitochondrial dysfunction

**Fig. 4:**
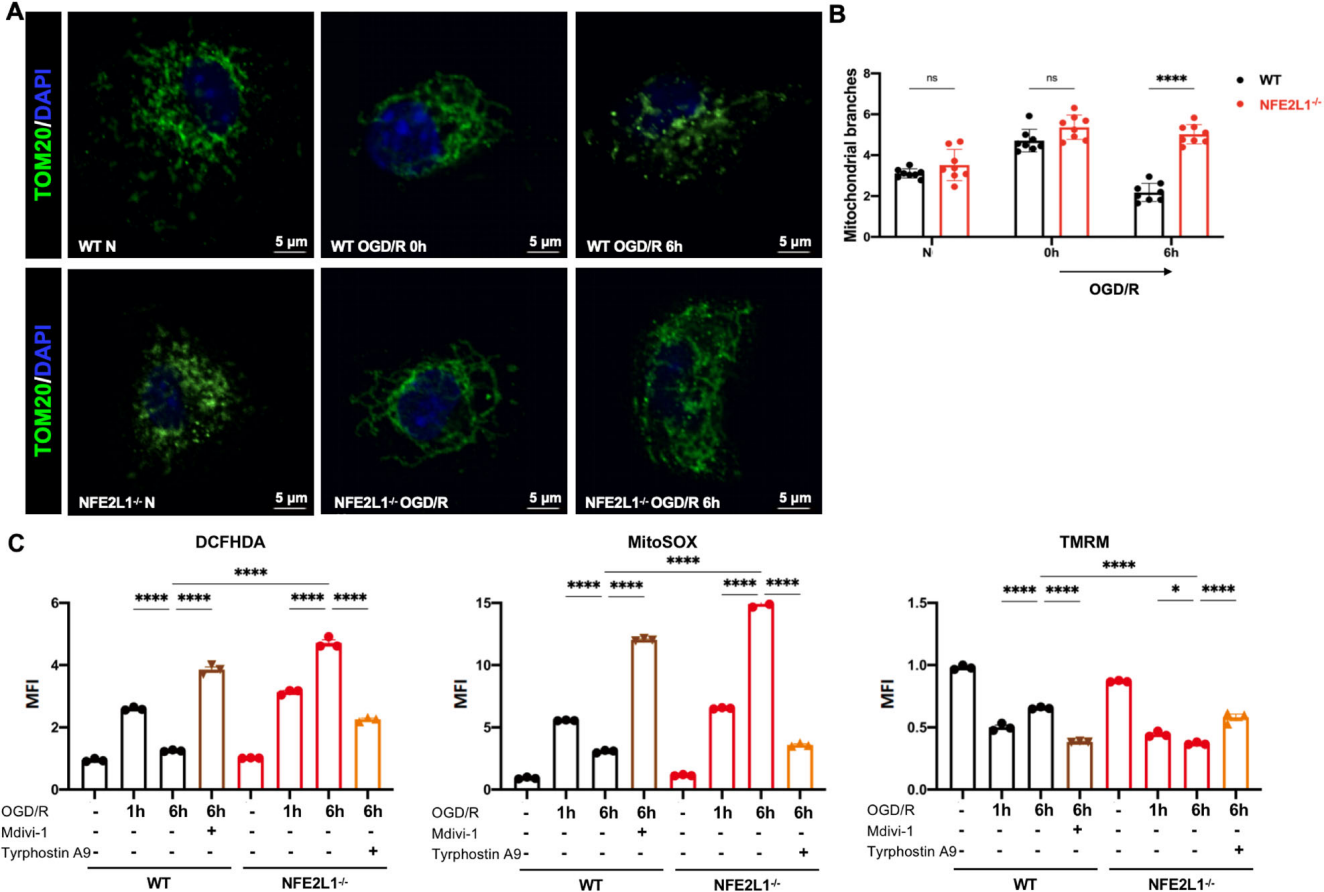
NRF1 deficiency increases mitochondrial fusion, ROS production and mitochondrial dysfunction. **A**, **B** TOM20 staining of WT and NFE2L1 KO BMDMs and visualization by immunofluorescent confocal microscopy. Scale bar = 5 μm. Representative image in A; analysis and quantitation by Image J in (**B**). **C** WT and NFE2L1 KO BMDMs were subject to OGD/R +/− Tyrphostin A9 (10 μM) or Mdivi-1 (30 μM) during reoxygenation. DCFHA, MitoSox and TMRM staining was assessed by flow cytometry and MFI shown normalized to the untreated condition. Statistical test by two-way or one-way ANOVA (*n* = 3–8 and representative of 3–8 independent experiments, n.s. no significance, *P < 0.05, **P < 0.01, ***P < 0.001, ****P < 0.0001).

To interrogate the role of NRF1 during OGD/R, we utilized mice with myeloid-specific knockout of the *Nfe2l1* gene that encodes NRF1. BMDMs from these mice displayed the expected loss of NRF1 protein (Supplementary Fig. 3). First, we examined fission-fusion dynamics in WT and Nfe2l1-deficient BMDMs. OGD/R stimulated mitochondrial fusion followed by fission in WT BMDMs (Fig. 4A, B), as was previously observed (Fig. 1D, E). However, Nfe2l1-deficient BMDMs underwent mitochondrial fusion in response to oxygen-glucose deprivation but failed to shift to mitochondrial fission upon reoxygenation (Fig. 4A, B). As discussed above, failure to undergo mitochondrial fission during reoxygenation could promote mtROS production and exacerbate mitochondrial damage. Indeed, we found that in WT BMDMs, OGD/R induced a transient increase in cellular and mitochondrial ROS and decrease of mitochondrial membrane potential, evident at 1 h after reoxygenation but returning back to levels observed in unstimulated BMDMs at 6 h of reoxygenation (Fig. 4C). In Nfe2l1^−/−^ BMDMs, such increases in cellular and mitochondrial ROS and decreases in membrane potential were sustained, being exaggerated at 6 h relative to 1 h of reoxygenation. These findings support the notion that the ability to upregulate mitochondrial fission during reoxygenation limits ROS production and maintains mitochondrial functionality. In further support, treatment of WT BMDMs with Mdivi-1 to promote mitochondrial fusion robustly increased cellular and mitochondrial ROS levels while decreasing mitochondrial membrane potential. Conversely, treatment of Nfe2l1^−/−^ BMDMS with Tyrophostin A9 to trigger mitochondrial fission alleviated the increased cellular and mitochondrial ROS levels and decreased mitochondrial membrane potential, restoring them to near WT levels. Together, these findings support the notion that during OGD/R, WT macrophages upregulate adaptive mechanisms—including mitochondrial fission—to curb ROS production thus preserving mitochondrial functionality. In contrast, failure to upregulate these adaptive mechanisms in NRF1 deficiency leads to hyperfused mitochondria, increased ROS, and mitochondrial dysfunction.

### NRF1 induces mitophagy during OGD/R

**Fig. 5:**
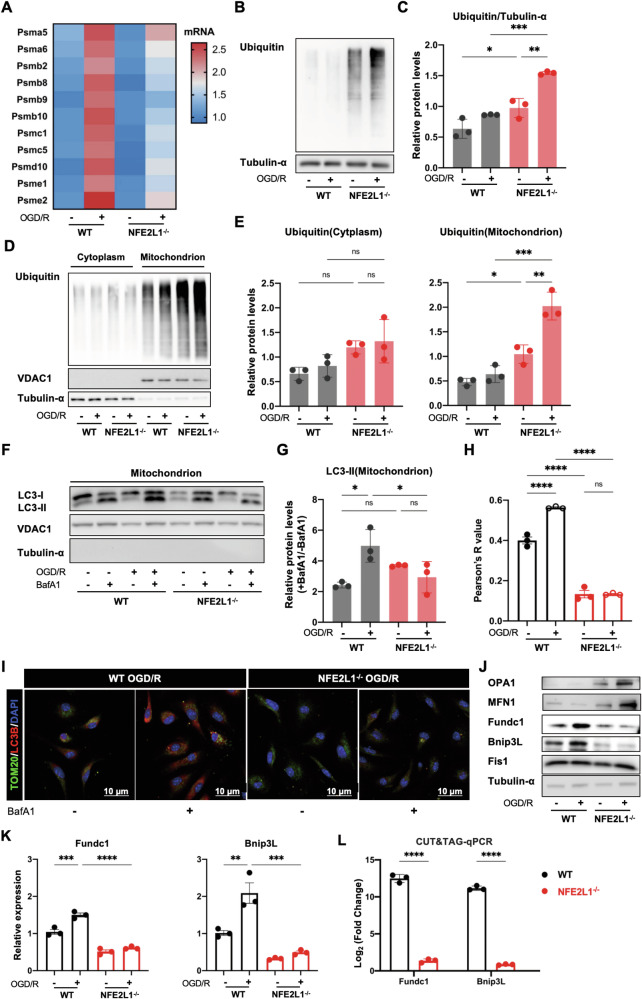
NRF1 induces mitophagy during OGD/R. **A** Heatmap indicating relative expression levels of proteasome subunit genes differentially expressed between WT and NFE2L1^−/−^ BMDMs subject to OGD/R. B-C. WT and NFE2L1^−/−^ BMDMs were subject to OGD/R followed by analysis of poly-ubiquitinated proteins by WB (**B**). Intensityof Ubiquitin was normalized to α-Tubulin (**C**). **D**, **E** WT and NFE2L1^−/−^ BMDMs were subject to OGD/R followed by analysis of NRF1 in cytosolic and mitochondrial fractions by WB (**D**). Intensity of Ubiquitin was normalized to α-Tubulin in cytoplasm and VDAC1 in mitochondria (**E**). **F**, **G** WT and NFE2L1^−/−^ BMDMs were subject to OGD/R +/− Bafilomycin A1 (1 nM) treatment during reoxygenation. LC3 lipidation in mitochondrial fractions was assessed by WB (**F**). Data are presented as fold induction of LC3-II band intensity in the +BafA1 condition versus the -BafA1 condition for the corresponding OGD/R stimulation time point (**E**). **H**, **I** WT and NFE2L1^−/−^ BMDMs were subject to OGD/R +/− Bafilomycin A1 (1 nM) treatment during reoxygenation. LC3 puncta and mitochondria co-localization was assessed by immunofluorescent microscopy. Quantitation (**H**) and representative images (**I**) are shown. **J**, **K** WT and NFE2L1^−/−^ BMDMs were subject to OGD/R followed by analysis of the indicated mitophagy regulators by WB with (**J**) and qRT-PCR (**K**). **L** WT and NFE2L1^−/−^ BMDMs subject to OGD/R were analyzed for NRF1 binding to the promoters of the indicated genes by Cut&Tag-qPCR. Statistical test by two-way ANOVA (*n* = 3 and representative of 3 independent experiments, n.s. no significance, *P < 0.05, **P < 0.01, ***P < 0.001, ****P < 0.0001).

We next examined proteasome subunit gene expression in WT and Nfe2l1^−/−^ BMDMs. While OGD/R triggered induction of several proteasome subunits in WT BMDMs, Nfe2l1-deficiency attenuated such induction (Fig. 5A). Moreover, Nfe2l1^−/−^ BMDMs accumulated higher levels of ubiquitinated proteins during OGD/R, relative to WT BMDMs (Fig. 5B, C). Biochemical fractionation studies indicated that during OGD/R, poly-ubiquitinated proteins were enriched in mitochondrial fractions, and that Nfe2l1^−/−^ BMDMs accumulated more poly-ubiquitinated proteins at the mitochondria compared to WT BMDMs (Fig. 5D, E). Together these findings indicate that during OGD/R, mitochondrial proteins are a target of the UPS, and in Nfe2l1^−/−^ macrophages, failure to upregulate proteasome gene expression leads to the accumulation of ubiquitinated proteins at the mitochondria.

To gain additional insight into the potential role of NRF1-mediated protein ubiquitination at the mitochondria, we performed RNA-seq of BMDMs subject to OGD/R. We found 423 upregulated and 317 downregulated genes during OGD/R (relative to unstimulated controls) (Supplementary Fig. 4A), and Mitophagy as one of the top enriched pathways by KEGG enrichment analysis (Supplementary Fig. 4B) This was of interest because mitophagy is an important mechanism to remove damaged mitochondria in conditions of cellular stress, and recent studies indicate that proteasome activity at the mitochondria can directly promote mitophagy. Specifically, ubiquitination and degradation of the mitochondrial fusion regulator MFN1 allows for mitochondrial fission and mitophagy [13, 30]. Examining LC3 lipidation in mitochondrial fractions to determine mitophagy flux, we found that mitophagy was induced by OGD/R in WT BMDMs, but that such induction was attenuated in Nfe2l1^−/−^ BMDMs (Fig. 5F, G). Co-localization of LC3 puncta with mitochondria also supported reduced mitophagy flux in Nfe2l1^−/−^ BMDMs (Fig. 5H, I). Furthermore, examining a panel of proteins that regulate mitophagy directly or indirectly, we found that levels of MFN1, and of another mitochondrial fusion regulator OPA1, were higher in Nfe2l1^−/−^ BMDMs subject to OGD/R, compared to WT counterparts (Fig. 5J, Supplementary Fig. 4C). In contrast, levels of the fission regulator FIS1 were comparable between WT and Nfe2l1^−/−^ BMDMs (Fig. 5J, Supplementary Fig. 4C). These findings are consistent with NRF1-dependent turnover of mitochondrial fusion proteins during OGD/R, likely dependent on proteasome activity.

In this analysis, we also found marked, NRF1-dependent induction of some key regulators of mitophagy. Specifically, OGD/R stimulation increased levels of FUNDC1 and BNIP3L in WT but not Nfe2l1^−/−^ BMDMs (Fig. 5J, K, Supplementary Fig. 4C). Because NRF1 is a transcription factor, we posited that it could upregulate the expression of Fundc1 and Bnip3l. Indeed, we found that NRF1 binds to promoter regions of these genes by Cut and Tag analysis (Fig. 5L, Supplementary Fig. 4D, E), indicating that NRF1 is recruited to these genes to mediate their induction.

In summary, this line of investigation shows that NRF1 activity induces mitophagy during OGD/R, likely contributing to mitochondrial quality control. Mechanistically, this may be mediated by upregulation of proteasome activity to promote mitophagy, as well as direct induction of some mitophagy regulators.

### Myeloid NRF1 curbs inflammation and tissue damage in IRI

**Fig. 6:**
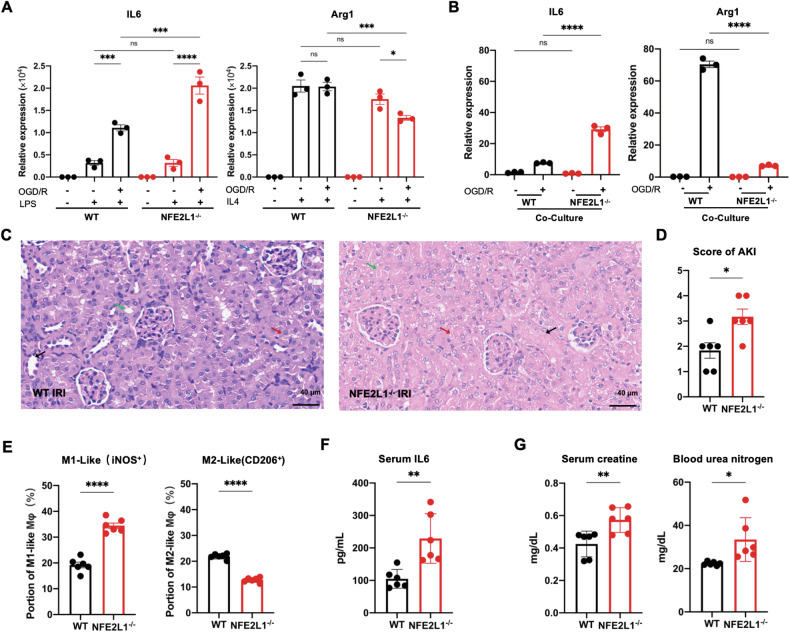
Myeloid NRF1 curbs inflammation and tissue damage in IRI. **A** WT and NFE2L1^−/−^ BMDMs were treated with OGD/R + /- LPS (10 ng/ml) or IL-4 (20 ng/ml), followed by analysis of Il6 and Arg1 gene expression by qRT-PCR. **B** WT and NFE2L1^−/−^ BMDMs were co-cultured with renal tubular epithelial cells during OGD/R, followed by analysis of Il6 and Arg1 gene expression by qRT-PCR. C-G. WT and mye-NFE2L1 KO mice were subject to IRI-AKI. **C** Kidney tissue sections visualized using hematoxylin-eosin (HE) staining. Blue arrow, normal renal tubules; black arrow, brush margin; red arrow, necrosis; green arrow, renal epithelial casts. **D** Kidney damage score: 0, no damage; 1, < 25%; 2, 25 to 50%; 3, 50 to 75%, 4, > 75%. **E** The proportion of iNOS^+^ and CD206^+^ cells amongst kidney F4/80^+^ cells was determined by flow cytometry. **F** Serum levels of IL-6 as detected by ELISA. **G** Serum creatine (Jaffe’s method) and nitrogen (Jung’s method) were measured by spectrophotometer. Statistical test by one-way ANOVA and 2-tailed samples t-test (*n* = 3–6 and representative of 3–6 independent experiments, n.s. no significance, *P < 0.05, **P < 0.01, ***P < 0.001, ****P < 0.0001).

We next asked how NRF1 activity influenced inflammatory responses during OGD/R. First, we induced BMDM activation to M1-like and M2-like phenotypes using LPS and IL-4 stimulation respectively. We found that NRF1 deficiency had no effect on such activation, as indicated by comparable expression of LPS-inducible Il6 and IL-4 inducible Arg1 in WT and Nfe2l1^−/−^ BMDMs. Next, we induced BMDM activation to M1-like and M2-like phenotypes under OGD/R stimulation. We found that, compared to WT counterparts, Nfe2l1^−/−^ BMDMs expressed higher levels of LPS-inducible Il6 but lower levels of IL-4-inducible Arg1 (Fig. 6A). Therefore, while NRF1 may not directly influence macrophage activation, it may modulate macrophage activation during OGD/R, presumably by regulating oxidative stress (see Discussion).

As another in vitro model to simulate macrophage activation during I/R, we co-cultured WT or Nfe2l1^−/−^ BMDMs with renal tubular epithelial cells (RTECs) during OGD/R treatment, reasoning that RTECs may provide signals to macrophages that influence their M1-like and M2-like activation. Indeed, we found that WT BMDMs could upregulate Il6 and Arg1 in such coculture conditions, and compared to WT BMDMs, Nfe2l1^−/−^ BMDMs made higher levels of Il6 and lower levels of Arg1 (Fig. 6B).

Collectively this line of in vitro experiments supports the notion that macrophage NRF1 deficiency may promote inflammatory responses during I/R. We next examined this hypothesis in a mouse model of IRI, subjecting WT mice and mice with myeloid cell-specific knockout of Nfe2l1 to IRI-AKI. In the sham condition, hematoxylin and eosin (HE) staining results of kidney sections revealed no significant difference between the two genotypes (data not shown). However, in the IRI condition, mye-Nfe2l1^−/−^ kidneys exhibited more severe injury (Fig. 6C, D). No normal tubular structure could be detected, and tubules were narrower and lacked a brush margin. Mye-Nfe2l1^−/−^ kidneys also displayed more disordered tubular epithelial cells, severe edema, and diffuse tubular epithelial vacuoles, and necrosis of multiple tubular epithelial cells resulted in formation of renal epithelial casts that were not observed in WT kidneys. Increased tissue injury in mye-Nfe2l1^−/−^ mice was associated with a higher proportion of kidney macrophages that were iNOS^+^ but a lower proportion that were CD206^ +^ , relative to WT counterparts (Fig. 6E). Furthermore, mye-Nfe2l1^−/−^ mice had higher serum levels of IL-6 (Fig. 6F, G), as well as higher serum levels of creatinine and urea nitrogen. Together, these findings indicate that myeloid expression of NRF1 plays a crucial protective role in renal injury.

## Discussion

**Fig. 7:**
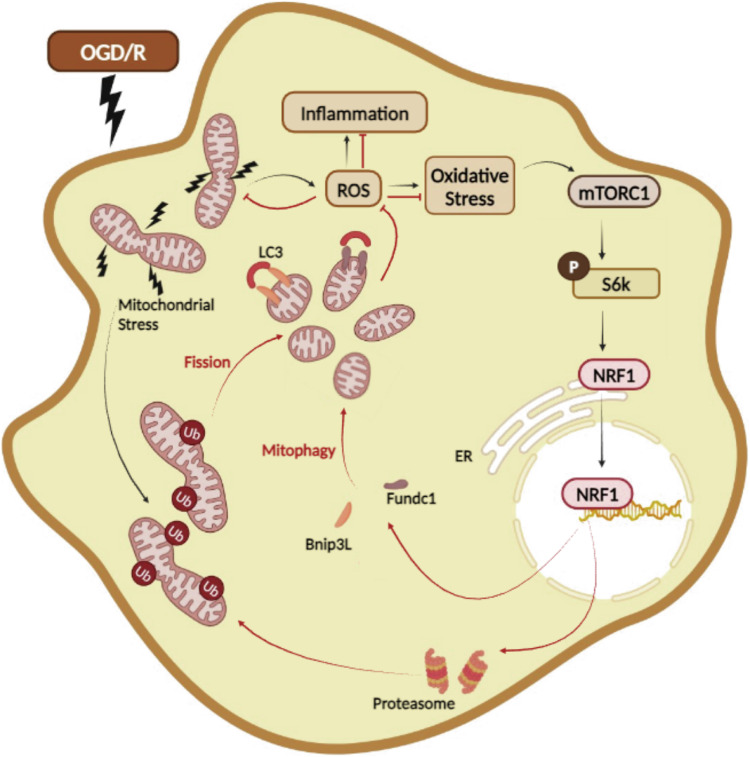
Working model. OGD/R triggers mitochondria to produce high levels of ROS, which activates the mTORC1 signaling pathway leading to the activation of NRF1. Activated NRF1 translocates to the nucleus, inducing genes regulating proteasome subunits to promote mitochondrial fission, which reduces mtROS production and facilitates mitophagy. Activated NRF1 also upregulates genes encoding mitophagy regulators to directly drive mitophagy. Thus, NRF1 coordinates mitochondrial adaptations that preserves mitochondrial integrity and reduces ROS levels and cellular stress. (Created with BioRender.com).

In this study, we exposed macrophages to OGD/R to elucidate how their inflammatory response is regulated during IRI. This model of OGD/R seemed to mimic IRI, given that it stimulated macrophages to undergo dynamic changes to mitochondrial dynamics and mtROS production, as well as inflammatory cytokine production. Using this model, we found that while OGD/R stimulation was insufficient on its own to promote inflammatory gene expression, it triggered ROS production and mitochondrial stress to potentiate inflammatory responses induced by LPS stimulation. Furthermore, we identified NRF1 as a master regulator of mitochondrial adaptations that curb detrimental ROS production during IRI. NRF1 responds rapidly to oxidative stress to upregulate a transcriptional program that promotes proteasome activity and mitophagy activity. Induction of proteasome activity supports mitochondrial fission to relieve mtROS production, while mitophagy activity maintains mitochondrial quality control. In this way, NRF1 coordinates mitochondrial adaptations that attenuate mitochondrial stress and mitochondrial damage to limit the macrophage inflammatory response (Fig. 7).

During OGD/R, production of ROS potentiates the macrophage inflammatory response. These findings are consistent with the ability of ROS to activate NF-kB and MAPK signaling, both of which contribute to the induction of IL-6 and other inflammatory cytokines [20, 31, 32]. Importantly, we found that mitochondrial fusion and fission are dynamically regulated during OGD/R to influence inflammation. Mitochondria fusion during the OGD phase is a response to nutrient deprivation, while rapid fission upon reoxygenation is an adaptive response to mitochondrial stress. Fission restricts mtROS production (by expanding the mitochondrial surface area and reducing membrane potential [24] to directly dampen mitochondrial stress, and also facilitates mitophagy to clear damaged mitochondria. Supporting a critical role for such regulation of mitochondrial dynamics, enforcing fusion or fission during OGD/R influences ROS levels and mitochondrial membrane potential to influence LPS-stimulated IL-6 production. To our knowledge, these findings are the first to show dynamic regulation of mitochondrial fission and fusion during OGD/R, as well as its non-redundant role in the associated inflammatory response.

NRF1 appears to be an important regulator of mitochondrial dynamics and mtROS production during OGD/R. NRF1 is rapidly induced in this context, entering the nucleus within 1–3 h of reoxygenation. Such NRF1 induction was regulated by mTORC1 signaling and influenced by the duration of OGD, suggesting that NRF1 is activated in proportion to the degree of oxidative stress. Once induced, NRF1 promotes proteasome activity to mediate clearance of ubiquitinated proteins, including ubiquitinated proteins at the mitochondria (Fig. 5D, E). Consistently, UPS flux is increased during reoxygenation, in a NRF1-dependent manner. Importantly, pharmacological interventions to inhibit proteasome activity or NRF1 deficiency led to increased mitochondrial stress, due at least in part to enhanced mitochondrial fusion (Figs. 3F–H, 4C). Previous studies showed that ubiquitination and proteasome-dependent degradation of mitofusins promote mitochondrial fission, and expression of MFN1 and OPA1 was increased in Nfe2l1^−/−^ BMDMs. Therefore, NRF1 activity links proteasomal degradation to mitochondrial fission and thus preservation of mitochondrial health during OGD/R. It is possible that degradation of additional mitochondrial proteins by the NRF1-proteasome axis, some of which are likely to be oxidatively damaged, could also contribute to mitochondrial health.

We also made the unexpected finding that NRF1 directly promoted mitophagy. In addition to increased levels of MFN1 and OPA1 in Nfe2l1^−/−^ BMDMs (likely due to impaired proteasomal degradation), we found that NRF1 was recruited to the promoters of mitophagy regulators including FUNDC1 and BNIP3L (Fig. 5J–L). At steady state, when mitochondrial stress and mitophagy flux are low, such defect in expression of mitophagy regulators do not appear to compromise mitochondrial morphology in Nfe2l1^−/−^ BMDMs. However, upon OGD/R-induced oxidative stress, NRF1-deficiency impairs mobilization of mitophagy (and proteasome gene expression) leading to exaggerated mitochondrial stress, relative to WT counterparts. A recent study found that NRF1 protects cardiomyocytes against IRI, attributed to NRF1 upregulation of proteasome expression and antioxidant defense [33]. Our study extends our understanding of the role of NRF1, by showing that the mitochondria are a major target of NRF1 activity in IRI, and that NRF1 deploys both UPS and mitophagy to coordinate mitochondrial homeostasis and dampen inflammation. Future studies to investigate the NRF1-mitophagy axis in regulation of mitochondrial quality control in other pathophysiological contexts, especially those characterized by oxidative stress and/or mitochondrial stress, are warranted.

In summary, our findings reveal that NRF1 is rapidly mobilized in macrophages during IRI to reinstate mitochondrial homeostasis and limit inflammation. Acute kidney injury caused by IRI is one of the most common complications in cardiac surgery [34], and myeloid deficiency of NRF1 led to elevated inflammatory responses and tissue damage after IRI-AKI surgery. Our study identifies NRF1 as a potential therapeutic target in IRI, and provides a rationale for the development of NRF1-directed agonists.

## Materials and methods

### Mice

All experiments utilized mice on C57BL/6 background. Mice with myeloid cell specific knockout of *Nfe2l1* (*Nfe2l1*^Δ/Δ^ mice) were generated by crossing *Nfe2l1*^fl/fl^ mice (GemPharmatech, strain T016349) and Lyz2-iCre mice (GemPharmatech, strain T03822). All mice were housed under specific pathogen-free conditions at ShanghaiTech University. Male and female mice were used for all experiments.

### BMDM culture and OGD/R

BMDMs from male and female mice were differentiated and cultured as described previously [35]. Randomization grouping is used in the experiment. To simulate ischemia-reperfusion injury, a glucose oxygen deprivation reoxygenation model was used. Cells were plated in glucose free 1640 RPMI medium (Gibco) and placed in a three gas incubator (Eppendorf) for 6 h of hypoxia treatment (37 °C, 5% CO_2_, 0.1% O_2_). After that, 10% FBS and 2 g/L glucose were added to the medium followed by incubation under normal oxygen conditions for 0–6 h before harvest. All drug treatments were added at the time of reoxygenation. Technical duplicates were analyzed for each cell sample.

### BMDM and TEC Co-culture

Renal cortex from the kidneys of C57BL/6 mice (6–8 week old) were subject to enzymatic dissociation with 1 mg/mL collagenases (Gibco) for 40 min. After filtering through a 70 µm cell filter, renal tubular epithelial cells (TECs) were obtained by centrifugation at 2500 RPM. TECs were placed in the lower chamber and BMDMs in the upper chamber of a Transwell chamber and OGD/R was initiated after cell adherence.

### qRT-PCR

Total RNA were collected in Trizol buffer (Vazyme) and isolated following an established protocol (Vazyme). cDNA was synthesized using 500 ng of total RNA using oligo (dT) primers and Reverse Transcriptase (Vazyme). Real-time qRT-PCR was performed in Bio-Rad CFX96 Touch real-time PCR detection system using Universal SYBr Green Supermix (Vazyme). Gene-specific primers sequences are listed in the supplementary table 1.

### Immunoblot and ELISA

For immunoblotting, BMDMs were lysed in RIPA buffer and protein concentration was determined using the Bradford method or Micro BCA Protein Assay Kit (ThermoFisher). The primary antibodies used for immunoblotting were as follows: anti-α-tubulin (1:3000, Abclonal, cat# A6830), anti-Ubiquitin (1:1000, CST, cat# 3936), anti-Lamin A/C (1:1000, Santa Cruz, cat# sc-376248), anti-NRF1 (1:1000, CST, cat# 8052S), anti-phospho-S6k (1:1000, CST, cat# 9234), anti-phospho-S6 (1:1000, CST, cat# 4858), anti-S6K (1:1000, CST, cat# 34475), anti-S6 (1:1000, Abclonal, cat# A11874), anti-OPA1 (1:1000, Abclonal, cat# A9833), anti-FIS1 (1:1000, Abclonal, cat# A19666), anti-MFN1 (1:1000, Abclonal, cat# A9880), anti-FUNDC1 (1:1000, Abclonal, cat# A16318), anti-BNIP3L (1:1000, Abclonal, cat# A6283), anti-LC3B (1:1000, CST, cat# 43566) and anti-VDAC1 (1:1000, Abclonal, cat#A19707). Then, the secondary antibodies we used were anti-mouse IgG(1:3000, Abclonal, cat# AS003) and anti-rabbit IgG(1:3000, Cell Signaling Technology, cat# 7074). Quantitation of the anti-Trex1 bands immunoblots was analyzed using ImageJ software (v 1.8.0). Intensities of bands were normalized to housekeeping proteins. For ELISA, IL-6 kits were from Biolegend.

### Ischemia and reperfusion injury

6–8 week old C57BL/6 mice were randomly divided into each group (*n* = 6). IRI-AKI was induced by clamping the renal pedicles for 35 min. The mice in the sham group received operations in which the kidneys were exposed but not clamped. After reperfusion for 1 and 24 h, mice were sacrificed. Serum was collected for analysis of serum creatinine (Bioassay), urea nitrogen (Bioassay), and IL-6 ELISA (Biolegend). Kidney tissue was embedded in paraffin for hematoxylin eosin (HE) staining and immunofluorescence staining. Tubular injury scores were calculated according to the percentage of damaged tubules: 0, no damage; 1, < 25%; 2, 25–50%; 3, 50–75%, 4, > 75% [36]. Blinding method was used for sample testing, and technical triplicates were analyzed for each sample.

### Cellular immunofluorescence of mitochondrial structure

Macrophages were cultured on glass slides, fixed with 4% PFA for 30 m, and incubated in blocking buffer (5% serum in 0.1% Triton X-100 PBS) for 1 h at room temperature. After staining with TOM20 primary antibody (1:200, Abcam, cat# ab283317) and primary antibody anti-LC3B (1:200, LC3B, cat#43566), fluorescent secondary antibodies (Alexa Fluor 488 anti-mouse IgG, Abcam, cat# ab150113 and Alexa Fluor 594 anti-rabbit IgG, Abcam, cat# 150080), and DAPI, the slides were sealed and observed under a fluorescence microscope. Co-localization was analyzed by ImageJ software(v 1.8.0).

### Cut & tag

Harvesting and of NRF1 (CST, cat# 8052S) binding-DNA and building DNA library were done following an established protocol (Novoprotein).

### Flow cytometry

Kidney tissue was mechanically dissociated using scalpels followed by enzymatic dissociation with 1 mg/mL collagenases (Gibco) for 40 min. Tissue fragments were filtered through a 70 µm cell strainer. Single cell suspension was stained with antibodies for iNOS (CST,cat# 48866), CD206 (Biolegend,cat# 141717), CD11b (Biolegend,cat# 101207), F4/80 (BD Biosciences, cat# 567201), NRF1 (CST, cat# 8052S) and Alexa Fluor 647 anti-rabbit IgG (Abcam, cat# ab150075) followed by flow cytometry analysis. For BMDMs, MitoSOX, MitoTracker Green and Red, TMRM and DCFDA staining were done according to manufacturer’s instructions (Invitrogen). Data were acquired with a FACS Calibur flow cytometer (BD Biosciences) and analyzed with FlowJo analytical software (v 10.4).

### Isolation of nuclear and mitochondrial proteins

Established protocols were used for isolation of nuclear (Beyotime Biotechnology) and mitochondrial (ThermoFisher) proteins.

### Statistical analysis

Mean ± s.d. is shown. Statistics were calculated using GraphPad Prism 9. Comparisons of two groups were analyzed using two-tailed t test, and comparisons of multiple groups were analyzed using one-way or two-way ANOVA as indicated. *P*-values < 0.05 were considered significant. In the figures, *, **, ***, and **** represent *P* ≤ 0.05, *P* ≤ 0.01, *P* ≤ 0.001, and *P* ≤ 0.0001, respectively.

## Supplementary information

Supplemental material

## Data Availability

The RNA-seq generated from this study have been deposited in the National Genomics Data Center under the project accession PRJNA1229881 and is publicly available. Any additional information required to reanalyze the data reported in this paper is available from the lead contact upon request.
