# Supplementary material for: NRF1 coordinates mitochondrial adaptations to dampen intracellular ROS and inflammatory responses during ischemia reperfusion
Source: Cell Death Discov. 2025 May 15;11:236. doi: 10.1038/s41420-025-02461-5 (PMC12081606; doi:10.1038/s41420-025-02461-5)
Supplement: Supplementary file 1 — Supplemental material [file 41420_2025_2461_MOESM1_ESM.pdf]

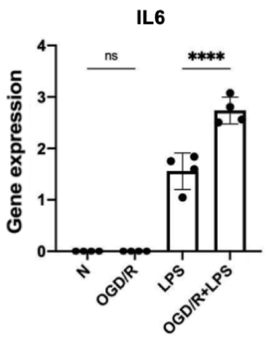

Supplementary Fig 1.

Il6 gene expression as assessed by qRT-PCR in BMDMs stimulated with OGD/R and LPS (20 ng/mL) as indicated. N, non-treated.

Statistical test by one-way ANOVA (n=4 and representative of 4 independent experiments, n.s. no significance, *P<0.05, **P<0.01, ***P<0.001, ****P<0.0001).

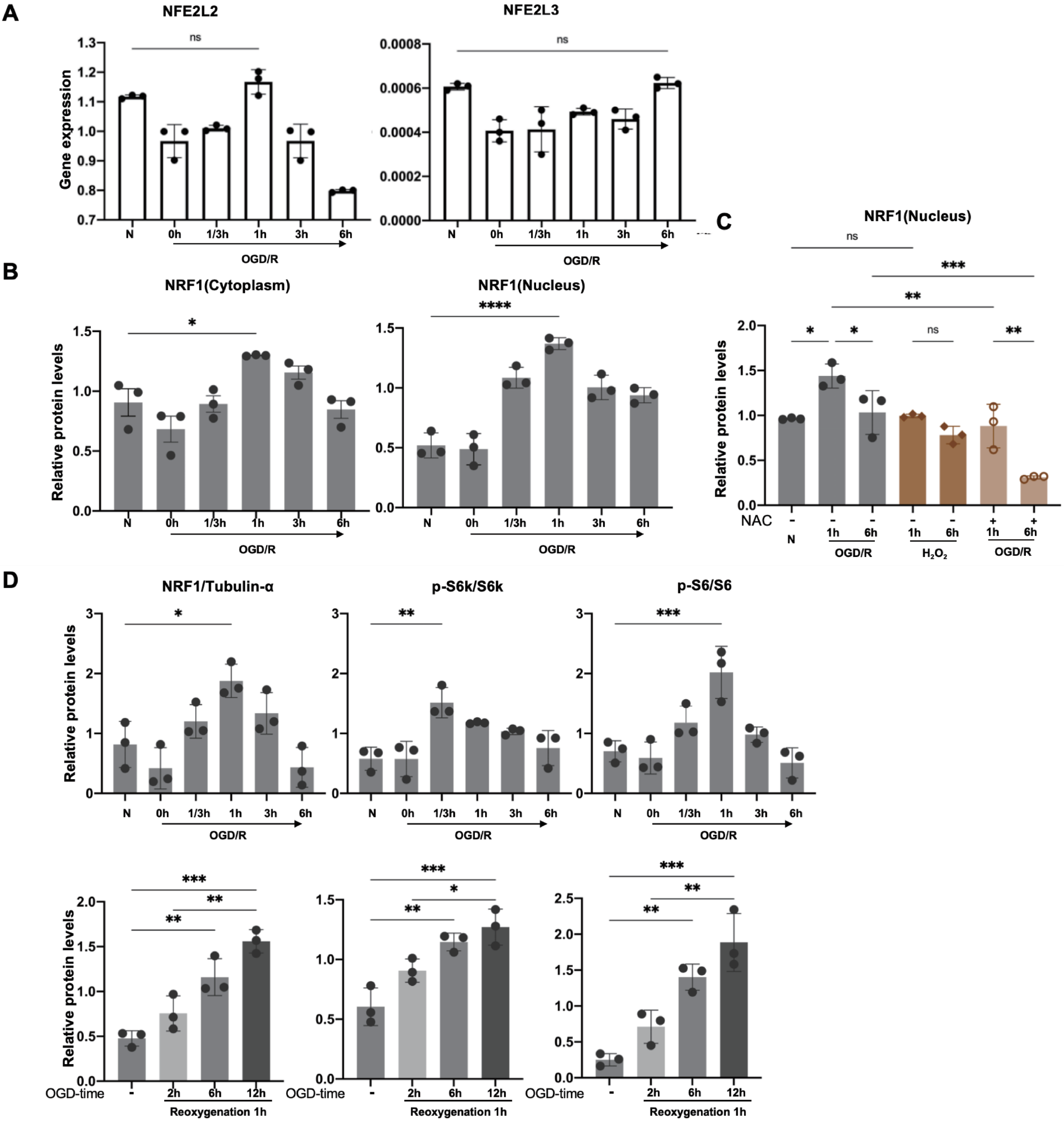

F

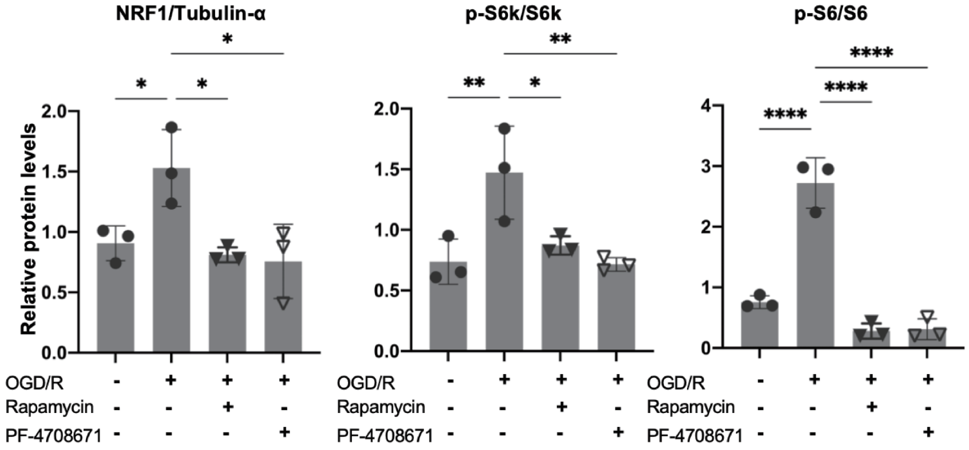

Supplementary Fig 2.

A.Gene expression of NFE2L2 (NRF2) and NFE2L3 (NRF3) in BMDMs subject to OGD/R.

B.BMDMs were treated with OGD followed by different durations of reoxygenation. NRF1 proteins levels in cytosolic and nuclear fractions was analyzed by WB, and quantitation of NRF1 was normalized to α-Tubulin in cytoplasm and LAMIN A/C in nucleus.

C.BMDMs were treated with OGD followed by different durations of reoxygenation +/- H_2O_2 treatment during reoxygenation. Quantitation of NRF1 proteins levels in nucleus is shown versus LAMIN A/C.

D.BMDMs were treated with OGD followed by different durations of reoxygenation. NRF1 protein levels and mTORC1 signaling were assessed by WB. Quantitative data are presented as relative protein levels of bands intensities including NRF1/α-Tubulin, pS6K/S6K, pS6/S6 for the corresponding OGD/R stimulation time point.

E.BMDMs were subject to different durations of OGD followed by 1h reoxygenation. NRF1 levels and mTORC1signaling were assessed by WB. Quantitative data are presented as relative protein levels of bands intensities respectively including NRF1/α-Tubulin, pS6K/S6K, pS6/S6.

F.BMDMs were subject to OGD/R +/- mTOR inhibitor (rapamycin, 400 nM) or S6K inhibitor (PF-4708671, 10 μM) during reoxygenation as indicated. NRF1 levels and mTORC1 signaling were assessed by WB(J). Quantitative data are presented as relative protein levels of bands intensities respectively including NRF1/α-Tubulin, pS6K/S6K, pS6/S6.

Statistical test by one-way ANOVA (n=3 and representative of 3 independent experiments, n.s. no significance).

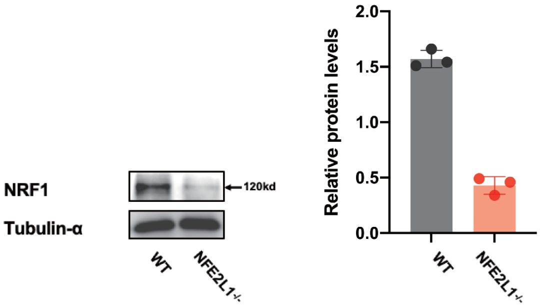

Supplementary Fig 3.

Whole cell lysates from WT and NFE2L1-/- BMDMs were assessed for levels of NRF1 by WB with quantitation, which was normalized to Tubulin-α.

Statistical test by one-way ANOVA (n=3 and representative of 3 independent experiments, n.s. no significance).

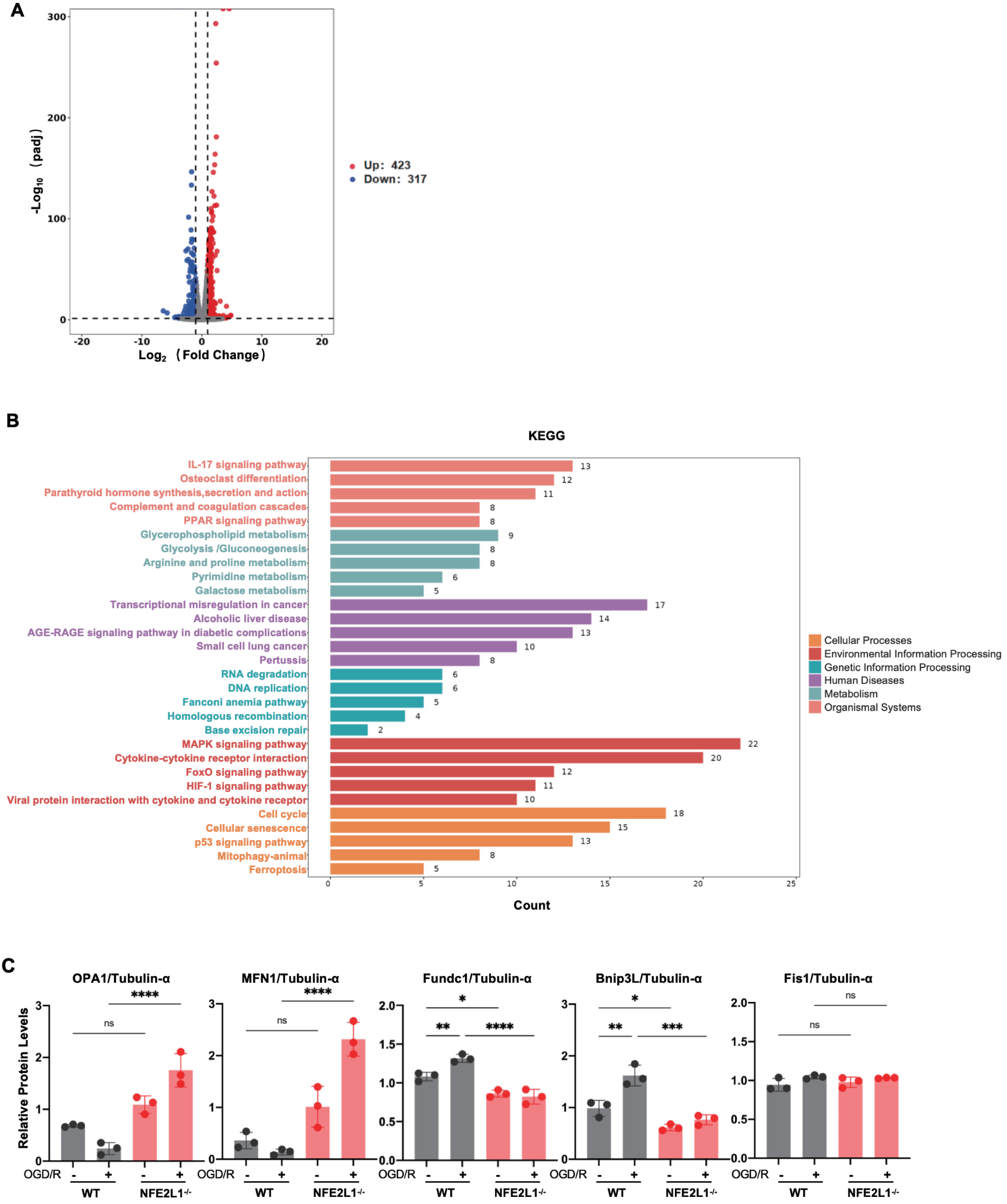
A
300
200
100
0
-20
-10
0
10
20
-Log10（padj）
Log2（Fold Change）
Up: 423
Down: 317
B
KEGG
IL-17 signaling pathway 13
Osteoclast differentiation 12
Parathyroid hormone synthesis,secretion and action 11
Complement and coagulation cascades 8
PPAR signaling pathway 8
Glycerophospholipid metabolism 9
Glycolysis /Gluconeogenesis 8
Arginine and proline metabolism 8
Pyrimidine metabolism 6
Galactose metabolism 5
Transcriptional misregulation in cancer 17
Alcoholic liver disease 14
AGE-RAGE signaling pathway in diabetic complications 13
Small cell lung cancer 10
Pertussis 8
RNA degradation 6
DNA replication 6
Fanconi anemia pathway 5
Homologous recombination 4
Base excision repair 2
MAPK signaling pathway 22
Cytokine-cytokine receptor interaction 20
FoxO signaling pathway 12
HIF-1 signaling pathway 11
Viral protein interaction with cytokine and cytokine receptor 10
Cell cycle 18
Cellular senescence 15
p53 signaling pathway 13
Mitophagy-animal 8
Ferroptosis 5
0 5 10 15 20 25
Count
Cellular Processes
Environmental Information Processing
Genetic Information Processing
Human Diseases
Metabolism
Organismal Systems
C
OPA1/Tubulin-α
MFN1/Tubulin-α
Fundc1/Tubulin-α
Bnip3L/Tubulin-α
Fis1/Tubulin-α
Relative Protein Levels
ns
****
*
**
***
OGD/R - + - +
WT
NFE2L1-/-

D

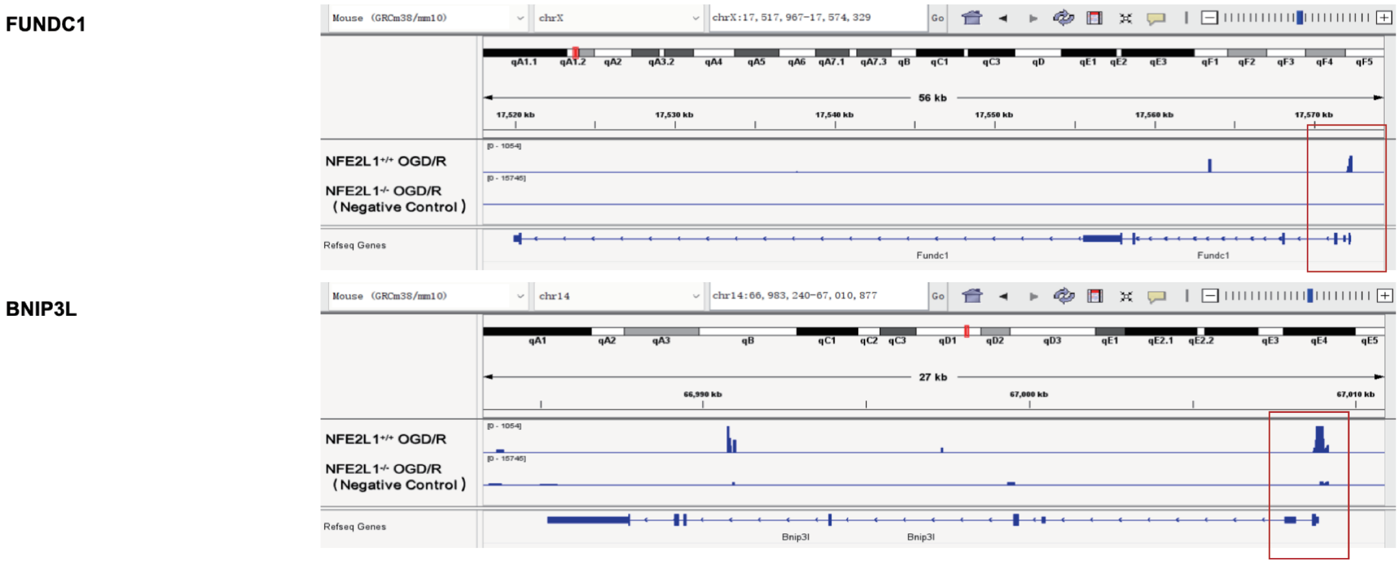

E

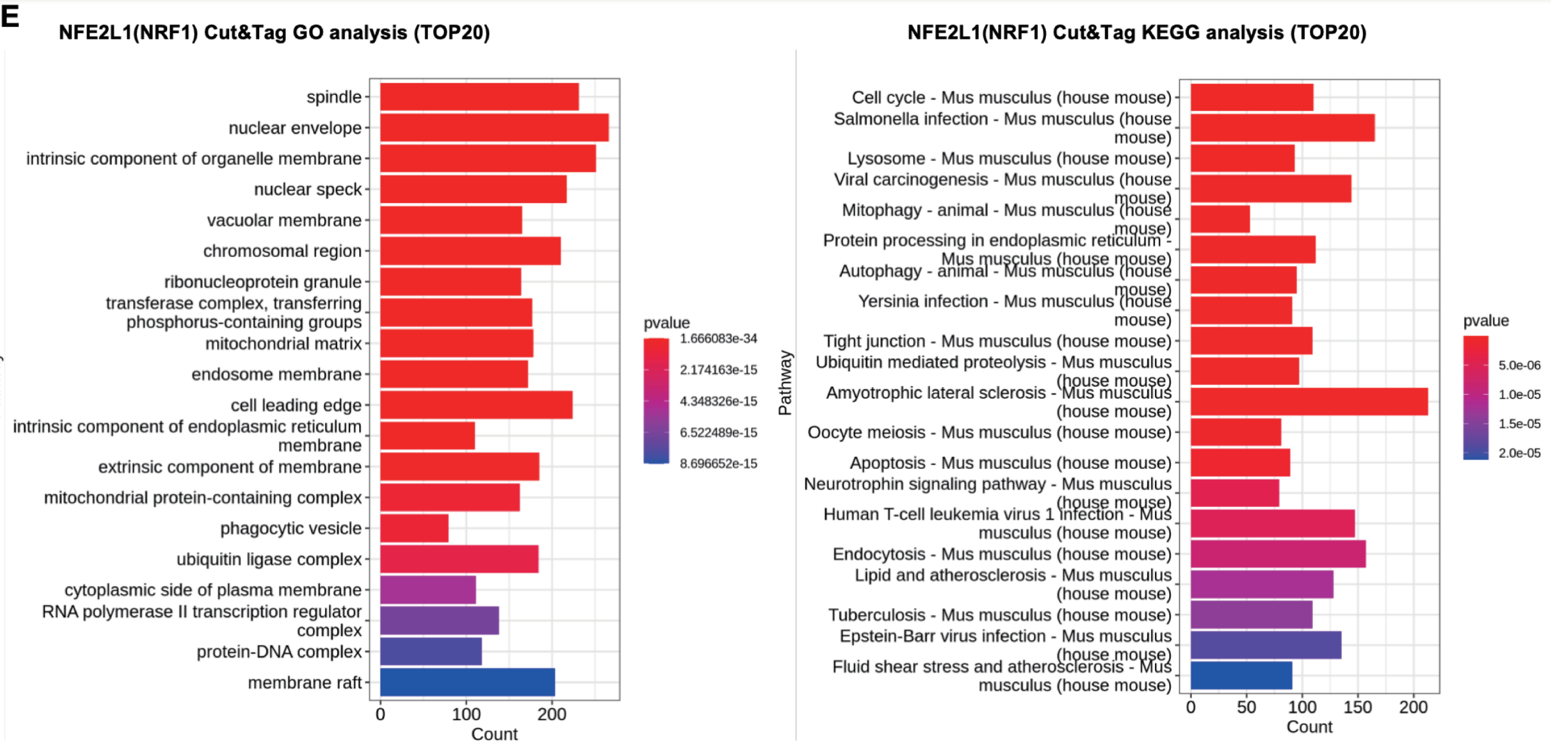

Supplementary Fig 4.

A-B. BMDMs were subject to OGD/R or not followed by RNA-seq analysis.

A. Genes up- and down- regulated by OGD/R stimulation relative to unstimulated.

B. KEGG analysis of gene upregulated by OGD/R stimulation relative to unstimulated.

C.WT and NFE2L1$^{-/-}$ BMDMs were subject to OGD/R followed by analysis of the indicated mitophagy regulators by WB with quantitation, which was normalized to Tubulin-α.

D-E. WT and NFE2L1$^{-/-}$ BMDMs were subject to OGD/R followed by NRF1 Cut and Tag-seq analysis.

D. Representative NRF1 tracks at the Fundc1 and Bnip3L genes displayed using IGV software.

E. GO and KEGG analysis to find enriched pathways in genes bound by NRF1 (i.e. upregulated in WT versus NFE2L1$^{-/-}$ BMDMs).

Statistical test by one-way ANOVA (n=3 and representative of 3 independent experiments, n.s. no significance).

Primers	5' to 3'
NFE2L1 forward	ACTGCAAGATGGACGAGAACGA
NFE2L1 reverse	GAAACGTCAGCTGGGAACTGTG
IL6 forward	ACAAAGCCAGAGTCCTTCAGAGAG
IL6 reverse	TTGGATGGTCTTGGTCCTTAGCCA
Psma5 forward	AATTGGCTCTGCTTCTGAGGGT
Psma5 reverse	GAAATTCTGACCAGGCTGCACC
Psma6 forward	AGGCCACTCGGTTGTTGTATGA
Psma6 reverse	AGCCACAGTAGTAACCTGCAGG
Psmb2 forward	ATGTGTTGGAGAGGCTGGAGAC
Psmb2 reverse	GATATGGGGTCCGACTCCGAAG
Psmb8 forward	TGGGGTGATGGACAGTGGTTAC
Psmb8 reverse	GCCTCTCCGTACTTGTACAGCA
Psmb9 forward	TATGGAACCATGGGAGGGATGC
Psmb9 reverse	GTGACCAGGTAGATGACACCCC
Psmb10 forward	TGTGGATGCCTGTGTGATCACT
Psmb10 reverse	GTGTGGTTCCAGGAGCAAATCG
Psmc1 forward	CTGCCGGATGAGAAGACCAAGA
Psmc1 reverse	CCAGAGAGGTCATCCTTTGCCA
Psmc5 forward	CACTGGGAAGACATTGTTGGCC
Psmc5 reverse	GTTCTCGGGCCATGACAAACAG
Psmd10 forward	GGTGCACATGTGAATGCTGTCA
Psmd10 reverse	TGGTCCTTCGCATCTGGGTTAG
Psme1 forward	GGTCACTACCTGGTTGCAGCTA
Psme1 reverse	ACTTGGAGATCTGCGTGTGGAA
Psme2 forward	GAGGCTGATGACTTCCTCTGCA
Psme2 reverse	GGAATCCTCCTGCAAGAGCTGA
Fundc1 forward	GTCGTGTACAGGGAAGAGTGG
Fundc1 reverse	TCTAGGCCGAGTTGGAAGTCT
Bnip3L forward	CTCAGCCCTTCCGGCTATTG
Bnip3L reverse	GTGCGGAAGTCGAGCTGATA
Actinβ forward	CGGTTCCGATGCCCTGAGGCTCTT
Actinβ reverse	CGTCACACTTCATGATGGAATTGA
NFE2L2 forward	CTTTAGTCAGCGACAGAAGGAC
NFE2L2 reverse	AGGCATCTTGTTTGGGAATGTG
NFE2L3 forward	TGAGCCAAGCTATAAGCCATGA
NFE2L3 reverse	AATGGTTCTTGTGCCTGTGAA

Supplementary table 1.
Primer sequences

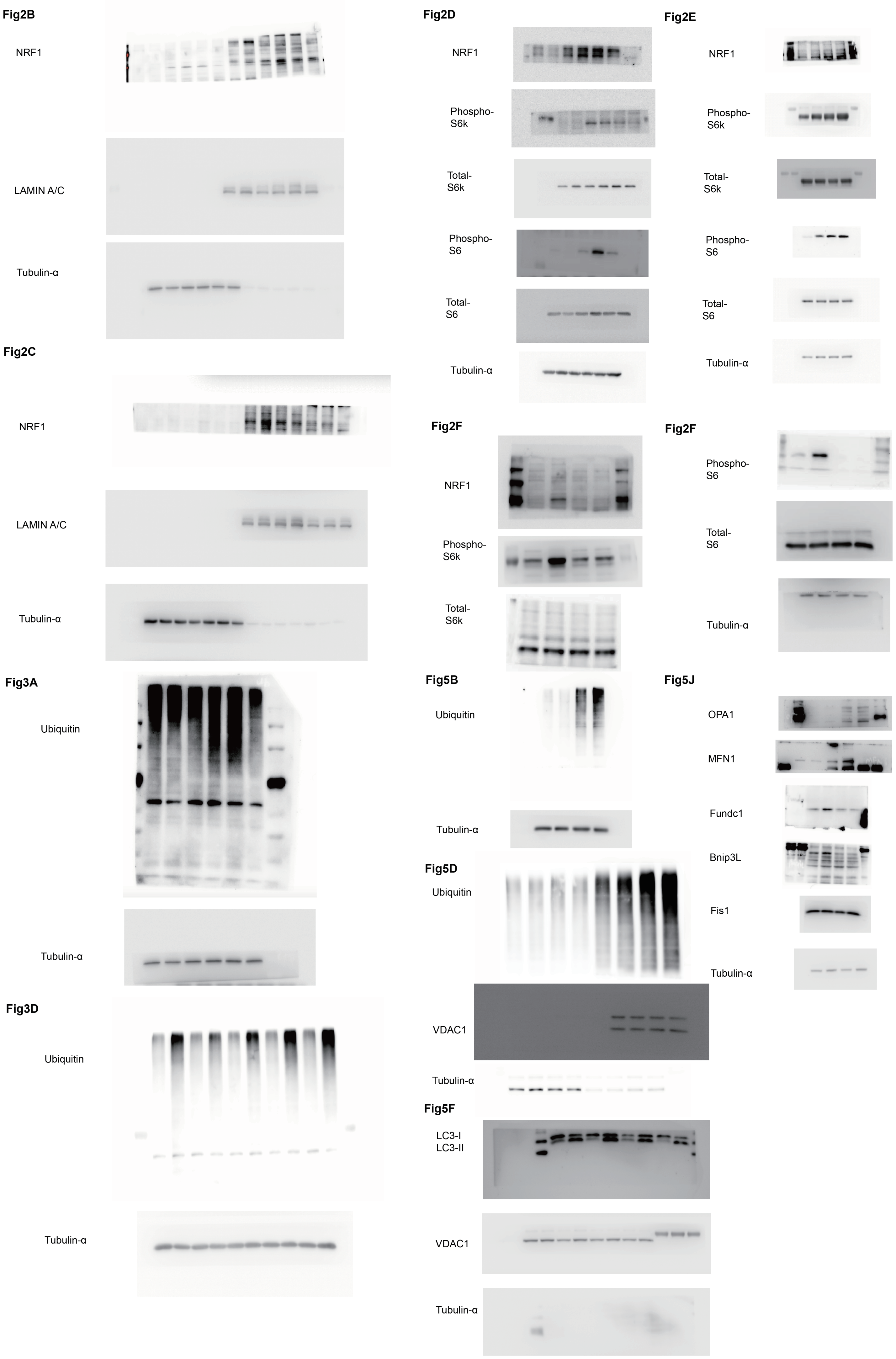

Main Figure full blot.

Supplementary Fig3

NRF1

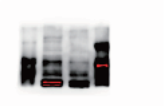

Tubulin-α

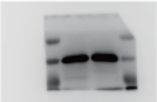

Supplementary Figure full blot.
